# Periostin and TNF-α expression levels in peripheral blood of patients with acute cerebral infarction combined with obstructive sleep apnea syndrome and their predictive value for clinical prognosis

**DOI:** 10.1186/s12883-022-02885-x

**Published:** 2022-09-20

**Authors:** Yu Xin, Shuai Li, Huimin Liu, Bo Liu

**Affiliations:** Department of Neurology, The First Affiliated Hospital of Baotou Medical College, Baotou, 014010 Inner Mongolia China

**Keywords:** Acute cerebral infarction, Obstructive sleep apnoea syndrome, Periostin, Tumour necrosis factor-α, Clinical prognosis

## Abstract

**Objective:**

To detect the expression levels of periostin and tumour necrosis factor-α (TNF-α) in patients with acute cerebral infarction (ACI) combined with obstructive sleep apnea syndrome (OSAS) and to investigate their predictive value for clinical prognosis.

**Methods:**

In this case‒control study, serum periostin and TNF-α levels were measured using ELISA, and patients were scored on the National Institutes of Health Stroke Scale (NIHSS) and modified Rankin Scale (mRS). Receiver operating characteristic curve(ROC) were generated to analyse the effect of peripheral blood periostin and TNF-α levels on poor prognosis.

**Results:**

NIHSS score, mRS score and peripheral blood periostin and TNF-α levels were higher in the observation group than in the control group (*P* < 0.001); serum periostin and TNF-α levels were positively correlated with the NIHSS score and mRS score (*P* < 0.001). Serum periostin and TNF-α levels were higher in patients with a poor prognosis than in those with a favourable prognosis (*P* < 0.001); the area under curve (AUC) values for the diagnosis of poor prognosis based on TNF-α, periostin or both factors were 0.868 (95% CI: 0.781–0.954), 0.834 (95% CI: 0.734–0.934), and 0.875 (95% CI: 0.792 ~ 0.958), with sensitivities of 0.654, 0.846, and 0.654 and specificities of 0.944, 0.750, and 0.917, respectively.

**Conclusion:**

Patients with ACI combined with OSAS have elevated peripheral blood periostin and TNF-α levels, and the combination of these two factors has high predictive value for poor prognosis.

## Background

Stroke is the primary cause of disability and death in adults worldwide, and its prevalence is rising [1]. The most common type of stroke is acute cerebral infarction (ACI), which accounts for 69.6 to 70.8% of strokes in China [2]. Obstructive sleep apnea syndrome (OSAS) is a condition in which the upper airway muscles collapse during sleep at night, partially or completely obstructing the airway [3]. In 2011, the American Heart and Stroke Association listed sleep apnea as a risk factor for the primary prevention of stroke [4], and the comorbidity rate of stroke and OSAS is as high as 70% [5]. The pathogenic mechanisms of OSAS, such as chronic intermittent hypoxia, sympathetic activation, changes in brain autoregulation, oxidative stress, systemic inflammation, a hypercoagulable state and endothelial dysfunction, increase the risk of stroke [6]. Patients with stroke combined with OSAS have severe neurological deficits, leading to prolonged hospitalization and rehabilitation and increased risks of stroke recurrence, morbidity and mortality [7]. TNF-α is a central element in the regulation of systemic inflammation and plays a vital role in ischaemia‒reperfusion injury caused by the rapid release of various cytokines [8]. Periostin is also an important proinflammatory cytokine in vivo. Both TNF-α and periostin can regulate each other and participate in a series of inflammatory responses in vivo that are involved in the pathogenesis of many diseases; moreover, these two factors may be potential biomarkers for assessing the severity of OSAS [9]. The correlation between periostin and TNF-α levels and the severity of OSAS in patients with ACI is unclear. This study aimed to investigate serum periostin and TNF-α levels in patients with ACI combined with OSAS and their relationship with prognosis for clinical reference.

## Materials and methods

### General data

One hundred and seven patients with ACI who were hospitalized in the Department of Neurology of the First Affiliated Hospital of Baotou Medical College, Inner Mongolia University of Science and Technology, between June 2021 and June 2022 were recruited. Inclusion criteria: (1) stroke symptom onset ≤ 72 h and brain tissue damage confirmed by cranial magnetic resonance imaging (MRI); and (2) ability to participate in completing the scales and neurological examination. Exclusion criteria: (1) history of malignancy; (2) acute or chronic infection; (3) autoimmune disease; (4) previous severe physical disability or mental illness; and (5) alcohol or drug dependence. The study was approved by the Ethics Committee of the Research Project of the First Affiliated Hospital of Baotou Medical College, Inner Mongolia University of Science and Technology (20210008), and all enrolled patients provided written informed consent.

### Grouping

All the patients underwent polysomnography (PSG) within one week of admission to determine whether they had combined OSAS, and the main observation indexes were apnea hypopnea index (AHI) and minimum oxygen saturation (LSaO2). The PSG device has Grael specifications and was produced by Comumedics, and the results were analysed by a practitioner with the relevant sleep study qualification. The patients were divided into the ACI combined with OSAS group (observation group) and the ACI alone group (control group) according to the sleep monitoring results. The observation group was divided into the good prognosis group and the poor prognosis group according to the mRS score.

### Research methods

#### Clinical data collection

General data were collected on admission, including demographic information (name, sex, age, height, weight, BMI, etc.), past medical history (hypertension, diabetes, heart disease, etc.), symptoms, and polysomnographic monitoring data.

#### Serum periostin and TNF-α levels and other blood indicators

Five millilitres of fasting peripheral venous blood was collected on the morning of the second day of admission. The samples were centrifuged at 1000 × g for 20 min after 2 h at room temperature, and the supernatant was extracted to detect serum periostin and TNF-α levels by enzyme-linked immunosorbent assay (ELISA) kits (Shanghai Jianglai Biotechnology Co) according to the kit instructions. Blood glucose, lipids and other relevant indicators were tested at the same time.

#### Severity assessment

Patients were assessed for ischaemic stroke severity using the National Institutes of Health Stroke Scale (NIHSS) within 24 h of admission.

#### Clinical prognosis assessment

The clinical prognosis of patients with neurological deficits was evaluated using the mRS on day 14 of disease onset, with patients scoring 0 to 2 placed in the good prognosis group and those scoring 3 to 6 placed in the poor prognosis group.

### Statistical analyses

Data with a normal distribution are presented as the mean ± standard deviation, and differences between groups were compared using the Student's t test; data that did not conform to a normal distribution are presented as the median (25th percentile, 75th percentile), and differences between groups were compared using the Mann‒Whitney U test. SPSS 20.0 statistical software was used for analysis. *P* < 0.05 was considered to indicate statistical significance.

## Results

### Comparison of general data

A total of 107 patients with ACI were included in the study. Sixty-two patients met the criteria for OSAS (observation group), and 45 patients did not meet the criteria (control group). There were no significant differences between these groups of patients in terms of age, sex or BMI. However, the diagnosis of diabetes was significantly more frequent in the observation group than in the control group, and blood glucose levels were significantly higher in the observation group than in the control group (*P* = 0.027). The NIHSS score and mRS score were higher in the observation group than in the control group (*P* < 0.001). The demographic data are summarized in Table 1.

**Table 1:** Demographic information for the two groups

Projects	ACI and OSAS group (*n* = 62)	ACI only group (*n* = 45)	*Test value*	*P value*
Sex [male, cases (%)]	40 (64.5)	27 (60.0)	2.636	0.621
Age (years)	61.68 ± 10.88	60.53 ± 12.71	0.500	0.618
BMI (kg/m^2^)	25.5 (23.9, 28.15)	24.8 (23.65, 27.3)	-1.196	0.232
Hypertension history [n, cases (%)]	52 (83.9)	34 (75.6)	1.143	0.285
Diabetes history [n, cases (%)]	16 (25.8)	4 (8.9)	4.910	0.027
Cardiac history [n, cases (%)]	7 (11.3)	5 (11.1)	0.001	0.977
Presence of neck plaques [n, cases (%)]	61 (98.4)	43 (95.6)	32.59	0.381
Blood glucose (mmol/L)	5.85 (5.00, 7.75)	5.1 (4.9, 5.5)	-3.423	0.001
Total cholesterol (mmol/L)	4.66 ± 1.04	4.53 ± 0.90	0.665	0.507
Triglycerides (mmol/L)	1.63 (1.31, 2.31)	1.43 (1.21, 1.94)	-1.174	0.240
High-density lipoprotein (mmol/L)	1.05 (0.92, 1.25)	1.11 (0.93, 1.28)	-0.761	0.447
Low-density lipoprotein (mmol/L))	3.03 ± 0.90	2.99 ± 0.82	0.238	0.812
Uric acid (µmol/L)	294 (232.25, 369)	324 (270, 397)	-1.660	0.097
Creatinine (µmol/L)	68 (53.75, 81.25)	73 (60.5, 84)	-0.761	0.447
AHI (times/h)	18.6 (12.45, 38.8)	3 (1, 4)	-8.770	< 0.001
LSaO_2_ (%)	81 (71.5, 85)	89 (87, 90.5)	-7.099	< 0.001
NIHSS score	4.5 (2, 9.25)	1 (1, 4)	-4.907	< 0.001
mRS score	2 (1, 4)	1 (1, 2)	-3.441	0.001

*Note*: Data that do not conform to a normal distribution are presented as the M (25th percentile, 75th percentile); data that conform to a normal distribution are presented as the mean ± standard deviation

### Comparison of serum periostin and TNF-α levels in the 2 groups

Serum periostin and TNF-α levels were significantly higher in the observation group than in the control group (*P* < 0.001) (Table 2).

**Table 2:** Comparison of serum levels of periostin and TNF-α in the 2 groups

Indicator	ACI and OSAS group (*n* = 62)	ACI only group (*n* = 45)	*Z*	*P value*
Periostin (ng/mL)	11.04 (10.49, 11.42)	8.49 (7.80, 9.23)	-8.654	< 0.001
TNF-α (pg/mL)	126.85 (100.73, 167.90)	69.4 (59.97, 77.82)	-8.514	< 0.001

### Correlation between serum periostin and TNF-α levels and NIHSS and mRS scores

Pearson correlation coefficient analysis showed that serum periostin and TNF-α levels were positively correlated with the NIHSS score and mRS score (*P* < 0.001), as shown in Table 3.

**Table 3:** Correlation between serum periostin and TNF-α levels and NIHSS and mRS scores

Indicator	NIHSS score		mRS score	
	***r***	***P***	***r***	***P***
Periostin	0.508*	< 0.001	0.571*	< 0.001
TNF-α	0.683*	< 0.001	0.677*	< 0.001

*Note*: *Indicates a significant correlation at the level of 0.01 (two-sided)

### Comparison of periostin and TNF-α levels in peripheral blood between the good prognosis group and the poor prognosis group

In the observational group, 58% of the patients (36 of 62) had a good prognosis (mRS score < 2), and 42% (26 of 62) had a poor prognosis (mRS score between three and six). Periostin and TNF-α levels in peripheral blood were significantly higher in the poor prognosis group than in the good prognosis group (*P* < 0.001) (Table 4).

**Table 4:** Comparison of serum TNF-α and periostin levels in patients with ACI and OSAS but with different prognoses

Group	n	Periostin (ng/mL)	TNF-α (pg/mL)
Good prognosis group	36	9.88 (7.90, 10.58)	130.84 (81.02, 178.37)
Poor prognosis group	26	11.12 (9.18, 11.53)	83.7 (69.4, 109.76)
***Z/P value***	-	-4.466/ < 0.001	-4.908/ < 0.001

### Ability of serum periostin and TNF-α levels to predict a poor clinical prognosis for ACI combined with OSAS

**Fig. 1:**
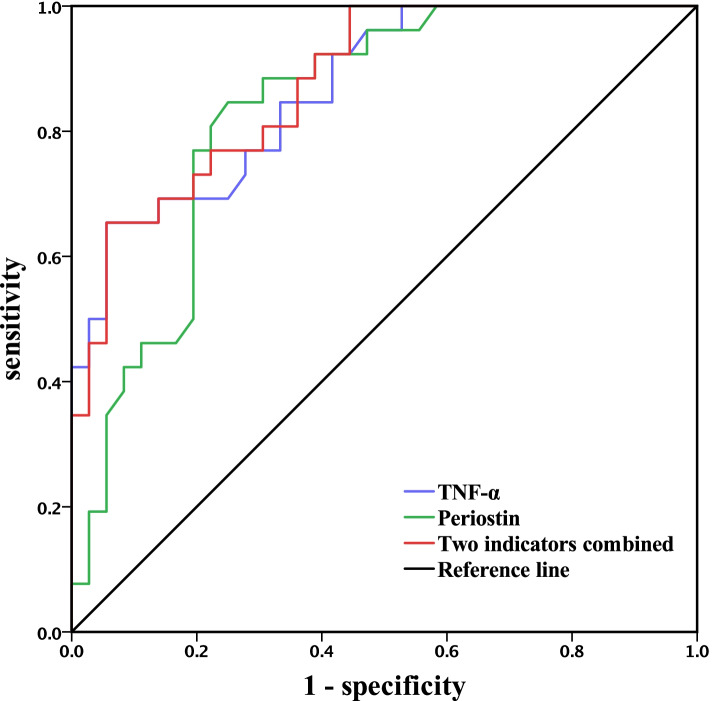
ROC curve of serum TNF-α and periostin levels to predict poor prognosis (*P* < 0.001)

Binary logistic regression established a combined predictive model of periostin and TNF-α, and the area under curve (AUC) of TNF-α, periostin and their combination for the ability to predict a poor prognosis was 0.868 (95% CI: 0.781 ~ 0.954), 0.834 (95% CI: 0.734 ~ 0.934), and 0.875 (95% CI: 0.792 ~ 0.958), respectively (Table 5 and Fig. 1).

**Table 5:** The prognostic value of the two factors for the diagnosis of ACI with OSAS

Indicator	*Optimal cut-off value*	*AUC*	*Sensitivity (%)*	*Specificity (%)*	*95% CI*	*P value*
Periostin	11.105	0.834	84.6	75	0.734 ~ 0.934	< 0.001
TNF-α	157.255	0.868	65.4	94.4	0.781 ~ 0.954	< 0.001
Periostin and TNF-α combined	-	0.875	65.4	91.7	0.792 ~ 0.958	< 0.001

## Discussion

OSAS is an independent risk factor for the development of stroke. OSAS causes a hypercoagulable state in the blood through chronic hypoxia and an inflammatory response, resulting in increased erythrocytes, platelet aggregation, and increased levels of fibrinogen and adhesion molecules [10]; these factors further aggravate ischaemia and hypoxia in brain tissue and damage vascular smooth muscle, thereby increasing the risk of stroke [11, 12]. The results of this study showed that the NIHSS score and mRS score were higher in the observation group than in the control group, indicating that the neurological deficits were more severe in ACI patients with combined OSAS.

Inflammation is a fundamental pathogenic factor in the development of atherosclerosis [13], and both periostin and TNF-α are important proinflammatory cytokines involved in the inflammatory response that can regulate each other to promote an inflammatory response [9]. Periostin is an extracellular matrix protein that regulates smooth muscle cells and foam cells and mediates atherosclerotic plaque formation [14], and increased serum periostin is thought to correlate with the severity of large atherosclerotic strokes [15]. Periostin has been shown to promote the proliferation and differentiation of neural stem cells in the brain after ischaemic or haemorrhagic injury, and in addition to exerting neuroprotective effects, increased serum periostin levels may promote microangiogenesis and prevent ischaemic injury after stroke [15, 16]. In this study, by comparing peripheral blood periostin levels in patients with ACI with and without coexisting OSAS, we found that peripheral blood periostin levels were significantly higher in patients with ACI combined with OSAS and were positively correlated with the NIHSS and mRS scores. The findings suggested more brain tissue damage in patients with ACI and OSAS compared to those with ACI alone, presumably through the binding of the fasciclin I structural domain of periostin to tenascin C (TNC) and subsequent coordinate regulation, causing blood‒brain barrier disruption [17]. Periostin may also activate the intracellular MAPK signalling pathway through integral proteins, and MAPKs induce and activate downstream matrix metalloproteinase-9 to degrade brain microvascular basal lamina and tight junction protein-1, leading to blood‒brain barrier disruption and brain oedema formation [18]. The former allows periostin to enter the circulation through the damaged blood‒brain barrier, causing elevated periostin levels in peripheral blood.

TNF-α is a proinflammatory cytokine with multiple biological activities that is involved in various pathological processes, such as the inflammatory response and immune response [19]. Intermittent hypoxia (IH) during sleep in OSAS patients is the main pathophysiological mechanism of systemic multisystem injury, leading to oxidative imbalance, increased oxygen free radicals, and the activation of proinflammatory cellular mediators such as hypoxia inducible factor-1α (HIF-1α), the most critical nuclear transcription factor that mediates the cellular hypoxic response; HIF-1 levels are influenced by the degree of tissue hypoxia. HIF-1α is activated during hypoxia and then promotes the transcription of its downstream target gene TNF-α, resulting in a series of stress responses to hypoxia [8, 20]. TNF-α also induces the production of reactive oxygen species (ROS), which drive the redox reactions that constitute "ROS signalling"; these ROS may also cause oxidative stress and lead to vascular dysfunction [21]. It has been shown that brain cell necrosis, increased blood‒brain barrier permeability, and increased TNF-α levels in focal areas of brain tissue after ACI lead to increased infarct size and brain damage [22]. The results of this study showed that peripheral blood TNF-α levels were significantly elevated in patients with ACI combined with OSAS and were positively correlated with the NIHSS and mRS scores, indicating that inflammation was aggravated by the occurrence of ACI and OSAS. In addition, intermittent hypoxia/reoxygenation (IHR) can also activate the TLR4/NF-κB signalling pathway, leading to inflammation and thus the upregulation of TNF-α, a downstream inflammatory factor [23]. TNF-α induces the expression of periostin through the transcription factor c-Jun [24], which is consistent with the results of the present study, in which Pearson correlation analysis showed that periostin was significantly positively correlated with TNF-α. Moreover, the current study showed that the release of periostin and TNF-α may trigger an inflammatory cascade response, thereby aggravating brain tissue damage. In addition, the AUC values for the ability of periostin and TNF-α to predict an adverse prognosis of ACI combined with OSAS were 0.834 and 0.868, respectively, indicating some predictive value for adverse prognosis. Further analysis showed that the AUC value for the 2 factors combined was 0.875; the predictive value of the combination was better than that of the individual factors. In addition, analysis of the relationship between periostin and TNF-α levels and the prognosis of ACI combined with OSAS showed that the incidence of poor prognosis was high among patients with high levels of periostin and TNF-α in peripheral blood, with severe neurological deficits and more serious consequences in the absence of timely treatment. The detection of peripheral blood periostin and TNF-α levels can help to determine and predict the condition and prognosis of patients with ACI combined with OSAS.

In summary, elevated peripheral blood periostin and TNF-α levels are observed in patients with ACI and coexisting OSAS and are associated with poor clinical outcomes. The measurement of periostin and TNF-α levels could be beneficial in predicting the outcome of patients with ACI and OSAS.

## Data Availability

All data generated and analysed during this study for this report are included in this published article. Additional study data can be requested from the corresponding author upon request.
